# Empowering bystanders: a psychological and institutional model for intervention in academic bullying

**DOI:** 10.3389/fpsyg.2025.1650438

**Published:** 2026-01-06

**Authors:** Debanjan Borthakur, Linda Crockett, Jenna Mihalchan, Jennifer Swann, Manob Jyoti Saikia

**Affiliations:** 1 Biomedical Sensors & Systems Lab, University of Memphis, Memphis, TN, United States; 2 Department of Psychology, University of Toronto, Toronto, ON, Canada; 3 Canadian Institute of Workplace Harassment and Violence Inc., Edmonton, AB, Canada; 4 Department of Biological Sciences, Lehigh University, Bethlehem, PA, United States; 5 Electrical and Computer Engineering Department, University of Memphis, Memphis, TN, United States

**Keywords:** psychology, bullying, academia, bystander, bystander action

## Abstract

“When you see something, say something”- is a familiar public-service slogan. Yet, in practice, speaking up can be stressful and carries significant individual risk. This paper examines the role of bystanders as critical actors in either perpetuating or disrupting harmful behaviors in the context of academic bullying. While previous research has primarily explored the experiences of targets and perpetrators in academia, this paper advances the discourse by introducing a conceptual model of bystander intervention in academic bullying. Drawing on Latané and Darley’s Five-Step Bystander Intervention Process, this model integrates the unique dynamics of power hierarchies, institutional culture, and professional risks that influence intervention choices in academia. This framework explains why bystanders often remain passive or, in some cases, align with the bully or bullies. Factors such as betrayal blindness, fear of reprisal, protection of self-interest, and the desire to maintain professional relationships contribute to bystander inaction or complicity. This paper concludes with recommendations and practical choices for institutional resources designed to strengthen accountability and foster a culture of psychological safety. By addressing the structural and psychological barriers that prevent bystanders from acting, our model provides a new pathway for fostering a safer, more inclusive, and ethically responsible academic environment.

## 1 Introduction

Academic bullying frequently centers on the misuse of power by those in positions of authority, leading to abusive supervision and may include abusing authorship and intellectual property rights, threatening to cancel funding or visas, and damaging the reputations of budding scientists via harmful recommendations or comments (Moss and Mahmoudi, 2021. Surveys find that academic bullying is widely prevalent. In a global survey of graduate students and postdoctoral researchers, 84% of researchers reported experiencing academic bullying, and 59% had witnessed it firsthand (Moss and Mahmoudi, 2021). The findings underscore that bullying is not an isolated incident but a pervasive, systemic issue within academia. While discussions on bullying have often focused on the perpetrator-target dyad, there is increasing recognition of the critical role played by bystanders, including colleagues, lab mates, students, staff, and other members of the academic community, who witness these incidents and influence whether harmful behaviors are perpetuated or addressed. Bystanders are the largest group exposed to bullying situations. Studies have reported that more than 80% of employees have witnessed workplace bullying (Pouwelse et al., 2021). Bystanders in academic settings can play a pivotal role; they may choose to a) support the target, b) remain silent, thereby enabling the abuse, or c) actively contribute to the harmful behaviors themselves (Thornberg et al., 2012). The bystander effect, a phenomenon describing how individuals are less likely to intervene when others are present (Darley and Latané, 1968) has been developed with inspiration from some incidents of bystander apathy. A similar example is the “Little Yue Yue” case in China, often cited as emblematic of public bystander apathy (Cheng et al., 2025). Institutions frequently lack clear policies and protective mechanisms that empower bystanders to take action. When systems consistently fail to support those who speak up, it becomes easier to understand why bystanders choose not to intervene. Recognizing these barriers allows the design of interventions that target documented impediments to bystander action (Lyons et al., 2025).

In this paper, first, academic bullying is defined in detail, and the role and type of bystanders are discussed. Although bystander intervention has been widely examined in K-12, campus safety, and general workplace contexts, its application to academic workplaces remains underdeveloped and fragmented. Existing guidance is often extrapolated rather than tailored to academic hierarchies, incentive structures, and governance. This paper introduces the Bystander Intervention Framework in Academic Bullying, grounded in Latané and Darley (1970) theory, but modified to incorporate the specific nature of bullying and the crucial influence of institutional dynamics unique to academia. Also, described the framework in steps, starting with the initial observation of bullying and concluding with the post-intervention feedback loop, and included a diagram to visualize the process. Next, the institutional and systemic barriers that often prevent well-intentioned bystanders from intervening are discussed, including the lack of *trauma-informed* training, passive or *laissez-faire leadership*, and *unconscious biases*. Finally, the last section of this paper highlights strategies to empower bystanders and implement institutional reforms. These strategies range from trauma-informed training programs and robust whistleblower protections to broader cultural shifts that promote what researchers refer to as institutional courage (Gómez et al., 2023). The conclusion consolidates the central argument that, while bystanders hold significant potential to intervene in academic bullying, realizing this potential requires both a shift in mindset and meaningful institutional change. Finally, this paper proposes directions for future research to test and refine the conceptual model presented in this study.

The model proposed here adapts Latané and Darley (1970) classic five-step bystander intervention framework to the unique context of academia, where bullying often thrives within rigid hierarchical structures. It explores how bystanders perceive and interpret incidents of academic bullying, and how institutional environments either facilitate or impede their willingness to intervene. Empowering bystanders requires addressing psychological and organizational barriers. On the psychological level, bystanders often experience fear, moral conflict, cognitive dissonance, and betrayal blindness, which inhibit their ability to act. Many suffer from “moral distress” when they recognize wrongdoing but feel powerless to intervene (Brüggemann et al., 2018). On the organizational level, factors such as unclear or ineffective reporting mechanisms, lack of institutional support, fear of reprisals, and perceptions of institutional betrayal further discourage bystander intervention (Brüggemann et al., 2018). To address these issues, implement comprehensive strategies that build supportive academic environments fostering *psychological safety* which is a shared belief that people can take interpersonal risks (speak up, ask questions, report mistakes, challenge ideas) without fear of punishment, humiliation, or retaliation- thereby enabling trust, candor, learning, and high team performance (Edmondson, 1999), for both the bystander and the target. If the environment fails to protect the target, it also fails those who may wish to intervene. This combination of psychological inhibition and institutional betrayal frequently leads to bystander inaction.

### 1.1 The role and typology of bystanders in academic bullying

Academic bullying is characterized by repeated, hostile behaviors in scholarly environments that undermine an individual’s dignity or career. This abuse, defined as “academic bullying,” is a sustained, hostile behavior from one’s academic superior, including, but not limited to, ridiculing, threatening, blaming, invasion of privacy, put-downs in front of others, as well as interference with matriculation and career progress including removing funding, writing falsely negative recommendation letters, taking credit for other’s work, and threatening to cancel visa or fellowships (Moss and Mahmoudi, 2021). Academic bullying commonly involves a power imbalance, such as a tenured professor targeting a junior faculty member or a doctoral student. However, bullying can also occur in a bottom-up dynamic, where subordinates target individuals in leadership positions. When subordinates engage in bullying behavior toward a leader, it often reflects a leadership issue, for example, the avoidance of conflict, a laissez-faire leadership style, or other barriers that prevent addressing challenging behavior. Further research is needed on the bullying of leadership to examine its causes, dynamics, and consequences, as well as to develop effective leadership strategies and institutional responses to address this complex and often overlooked form of workplace aggression. Multiple reports document underrecognition and weak responses to bullying in academic settings. Most institutions still lack clear, enforceable policies to prevent or address it, allowing harm to continue unchecked,” supported by Offer et al. (2023), Manuel et al. (2023), and Keashly and Neuman (2010), who document underreporting and weak institutional responses in academic settings. Academia is widely known as a fertile ground for workplace bullying, primarily because of its pronounced hierarchies. Power differentials between senior faculty and their dependents, such as graduate students, postdoctoral researchers, and early career professors, create circumstances for abusive conduct to flourish (Keashly and Neuman, 2010). Subordinates’ reliance on supervisors for funding, mentorship, reference letters and future opportunities, coupled with scant external oversight, worsens their vulnerability (Einarsen et al., 2011). Departmental leadership is often weak, and managerial approaches tend toward laissez-faire, leaving little accountability for inappropriate supervisory behavior (Branch et al., 2013). A fiercely competitive “publish-or-perish” culture can further legitimize bullying as a supposed performance driver (Salin, 2003). This is consistent with broader findings that excessive criticism, setting unrealistic targets, and repeated negative evaluations are among the most common mechanisms of workplace bullying (Bunce et al., 2024). Those with the least institutional power, graduate students, postdocs, junior faculty, women, and scholars from marginalized groups, bear the brunt of this dynamic (Keashly and Neuman, 2010). Targets of bullying are frequently the most vulnerable members of the scientific workforce, such as low-paid graduate students or postdocs, often living in foreign countries and navigating unfamiliar cultures (Mahmoudi and Keashly, 2021). A cross-sectional global survey conducted by Mahmoudi and colleagues (Moss and Mahmoudi, 2021) found that the age of perpetrators significantly influences the contextual behaviors of academic bullying, with younger perpetrators, aged 25–35, exhibiting less abusive behavior compared to older age groups (Moss and Mahmoudi, 2024). Moreover, academic bullying behaviors are discipline-specific, requiring targeted and nuanced approaches to addressing academic bullying (Moss et al., 2022; Täuber et al., 2022).

Witnesses do not uniformly experience harm. Some bystanders report distress and reduced engagement, whereas others maintain wellbeing and adopt defender-type roles, aided by personal resources (e.g., efficacy) and supportive climates. This section describes the phenomenon and distinguishes two bystander outcome paths: a) witnesses who experience psychological harm (e.g., distress, exhaustion) and b) those who do not, often engaging in constructive roles that mitigate harm. Table 1 maps common bystander roles onto these likely trajectories, providing a descriptive baseline for later explanatory and intervention. Bystanders are individuals who witness bullying but are neither the perpetrator nor the direct target. Drawing conceptually on Jönsson and Muhonen (2025), who examined how organizational context and situational strength shape bystander intentions to intervene, we adapt and extend their framework to the academic workplace. In this context, five broad bystander roles emerge: the *Silent Bystander, Complicit Participant, Witness with Separate Agenda, Hesitant Witness*, and *Active Defender*, each reflecting distinct motivational barriers and opportunities for intervention. For clarity, we group bystander roles into two outcome trajectories: higher-risk profiles (Silent Bystander, Complicit Participant, Witness with Separate Agenda, Hesitant Witness), which are more likely to be associated with distress and disengagement, and a lower-risk profile (Active Defender), which is more often linked to preserved wellbeing and constructive action, consistent with established bystander typologies and evidence on witnessed bullying and its effects (Paull et al., 2012; Salmivalli et al., 1996).

**TABLE 1:** Bystander roles, obstacles, practical actions, and likely outcomes.

Trajectory	Role	Typical obstacles	Practical actions (to shift toward constructive action)	Likely outcome
Higher risk	Silent bystander	Fear of retaliation; distrust of reporting channels.	Validate privately; encourage discreet documentation; seek a safe ally (e.g., union, ombudsperson); provide low-exposure options to act.	Potential harm: distress, moral injury, disengagement, unless protections & allies are established.
Higher risk	Complicit participant	Bystander(s) join the bully or mobbing group to protect status or fit in.	Surface long-term reputational costs; invite values check; model quiet dissent; build alternative alliances.	Potential harm: value conflict, reputational risk, impact on self-worth, self-respect, and self-confidence may be impacted.
Higher risk	Witness with a Separate Agenda	Leverages bullying to gain advantage; mobbing escalates.	Request conflict-of-interest review; push for transparent process, e.g., trauma-informed investigation and/or *trauma-informed* professional facilitation.	Potential harm: systemic fallout, backlash, unless incentives are realigned.
Higher risk	Hesitant witness	Wants to help but fears consequences.	Offer physical presence (co-witness); share secure reporting routes; set realistic boundaries; rehearse safe scripts.	Potential harm: anxiety, shame, guilt, unless safe actions are supported.
Lower risk	Active defender	Has leverage (e.g., tenure, status) but faces visibility costs.	Accompany the target while reporting; escalate through formal channels; raise public awareness strategically.	Non-harmed: agency preserved, monitor for burnout/retaliation risk.

All should commit to documenting whether they choose to report or not. This data will matter if and when they change their minds.

#### 1.1.1 Silent bystanders

Some people choose to be silent bystanders. Fear of retaliation or career harm is the single most reported barrier to intervention in longitudinal workplace surveys (Pouwelse et al., 2021). Learned helplessness from past failed complaints leads witnesses to expect institutional inaction. Physical or mental health strain (e.g., chronic illness, trauma) lowers the capacity to intervene. Many staff members are unsure of what constitutes evidence or where to submit it. Betrayal blindness also affects bystanders; it is “the unawareness, not-knowing, or forgetting exhibited by people in response to betrayal” and serves as a protective mechanism to preserve crucial relationships or institutional ties (Freyd, 1996). It extends to witnesses who depend on the perpetrator or the institution, fostering collective silence even when harm is obvious.

#### 1.1.2 Complicit participant

Joins in actively to avoid becoming the next target or to conform to group norms. They may reflect on long-term costs to culture and personal integrity. They might choose accountability by apologizing or expressing concern to the target in private. They can and should form alliances, as coordinated support makes retaliation less likely. Academic bullying thrives in step hierarchies: surveys suggest that almost half of researcher’s report being bullied, about two-thirds have witnessed it (Abbott, 2020).

#### 1.1.3 Witness with a separate Agenda

Leverages bullying to gain personal or factional advantage while they may amplify rumors, withhold information, or aligne with powerful actors to sideline the target. This behavior might accelerate mobbing and corrodes due process.

#### 1.1.4 Hesitant witness (higher risk)

Wants to help but fears retaliation, career damage, or social isolation; uncertainty about “what counts” as evidence and where to take it compounds paralysis.

#### 1.1.5 Active defenders

Defenders consciously recognize bullying and choose to act, a role documented across multiple workplace studies that chart “defender/helping” responses (Pouwelse et al., 2021). Moral courage and justice orientation are common drivers; experiencing bullying further strengthens this commitment (Holm et al., 2023). Typical actions of active defenders include directly confronting or interrupting the perpetrator to de-escalate the incident and signal norms against abuse. \Reminding targets of rights and resources, such as ombuds offices, unions, and HR codes, are effective approach when neutral, confidential, and trusted.

## 2 Institutional barriers and systemic issues preventing bystander action

### 2.1 Psychological Impact of bullying on bystanders

Bystanders who witness bullying frequently report elevated symptoms of anxiety and depression (Rivers et al., 2009). In fact, repeated exposure can lead even to emotional trauma (Carney and Hazler, 2011). Some research also suggests that uninvolved bystanders may show higher internalizing symptoms compared to non-witness peers (e.g., Juvonen et al., 2000). One striking real-world example is an independent review that exposed a deeply rooted culture of bullying, sexism, racial discrimination, and nepotism within the Australian National University’s (ANU) College of Health and Medicine. The investigation revealed that harassment and bullying were pervasive and often went unpunished (Nothling, 2025). Witnessing bullying likewise negatively impacts mental health and job satisfaction (Manuel et al., 2023), and bystanders may also experience moral distress (Brüggemann et al., 2018). Additionally, cognitive dissonance can explain their inaction, as they rationalize the bully’s actions- believing, for example, that “the student deserves the treatment,” a response akin to victim blaming (Garland et al., 2016). We further contend that performance appraisals also serve as tools to suppress any complaints. Thus, bystanders may also experience anger and frustration directed toward the organization (Johnson and Joshi, 2016). Witnessing academic bullying has been associated with anxiety, depression, and emotional exhaustion among faculty and trainees (Manuel et al., 2023). Some bystanders may even develop compassion fatigue (Köverová and Raczova, 2018) or empathic distress when witnessing a colleague being threatened or humiliated, which can potentially retraumatize them. Bystanders may emotionally “numb” themselves, reducing empathic response when the emotional burden becomes overwhelming (Hortensius and de Gelder, 2018). Moreover, some bystanders engage in moral disengagement, which refers to a set of cognitive mechanisms separating moral standards from actions, allowing engagement in unethical behavior (Moore, 2015). This disengagement may lead to a refusal to intervene. Passive or reinforcing behaviors have been linked to higher moral disengagement and lower empathy (Raboteg-Šarić and Bartaković, 2019; Xie et al., 2023). Moral rationalization is another term used by Tsang (2002) to describe a psychological phenomenon in which individuals convince themselves that their behavior does not violate their moral standards (Sereda, 2018, p. 30). Moral rationalization could partly explain the coping mechanism of both an aggressor and an inactive bystander. Empowering bystanders to intervene, therefore, has the potential to improve the overall mental health climate and productivity, benefiting not only the immediate target but the entire academic community.

A reason why bystanders remain inactive and are reluctant to intervene is a pervasive fear of institutional retaliation, a phenomenon known as institutional betraya**l**. Institutional betrayal occurs when an institution fails to protect individuals from harm or, worse, exacerbates the situation (Smith and Freyd, 2014). A study published in FEBS Letters, a journal of the Federation of European Biochemical Societies, highlights that challenging misconduct in academic settings more often results in retaliation than recognition (Täuber et al., 2022). Institutional betrayal can present in a number of ways, including ignoring or minimizing complaints; siding with or protecting perpetrators (cover-ups); retaliating against people who report or engage in protected activities; shifting blame onto the target or witnesses (victim-blaming); and other similar acts or omissions by the institution that fail to prevent or respond supportively to harm (Smith and Freyd, 2013, 2014; Pinciotti and Orcutt, 2021). Reported reasons for inaction include anticipated retaliation and low expected institutional support (Keashly and Neuman, 2010; Mahmoudi, 2019). It is also driven by a lack of institutional support: when staff expect complaints will be ignored, mishandled, or used against complainants, organizational silence follows and bullying stays unchecked (Morrison and Milliken, 2000; The Guardian, 2020; Adams-Clark et al., 2024). Institutional betrayal—protecting the bully to preserve reputation—amplifies harm and reduces future reporting; stronger accountability (not just promises of “courage”) is needed to deter misconduct (Freyd, 2023; Bloeser et al., 2023).

### 2.2 Lack of in-depth trauma-informed training in addressing academic bullying

Universities and workplaces typically offer training programs that define bullying in basic terms and explain institutional policies and reporting procedures (Escartín, 2016; Crusto et al., 2024). Although these programs are necessary and provide a foundational baseline, evidence shows they often deliver only short-term gains in knowledge or attitudes and frequently do not incorporate comprehensive, trauma-informed guidance that addresses the complex emotional and psychological realities of bullying and harassment (Htun et al., 2022; Kettrey and Marx, 2019). That is, they rarely foster heightened awareness of trauma’s impact or teach how to tailor responses accordingly (Canadian Institute of Workplace Bullying Resources [CIWBR], n.d.). A trauma-informed perspective is essential for recognizing subtle signs of distress, social exclusion, and patterns of escalating harm but it should also include recognition of indicators that appear *before* trauma develops. Equally critical is the need to recognize and respond to the early signs and symptoms that emerge before trauma sets in. Preventing trauma requires heightened awareness of the subtle behavioral shifts, emotional responses, and interpersonal dynamics that signal an individual is under psychological strain. These pre-trauma indicators, such as withdrawal, irritability, absenteeism, or fearfulness, are often overlooked or misattributed, yet they are red flags that demand immediate attention. When identified early, they offer a critical window for intervention. The trauma-informed training recommended in this section is designed precisely to support that prevention. Without this guidance, leaders, faculty, and staff are often ill-equipped to identify the multifaceted and nuanced behaviors characteristic of adult bullying. Academic environments are distinguished by power imbalances, prestige, and competition, factors that further reinforce the concealment, justification, and even normalization of abusive conduct.

Notably, bullying is often framed as a legitimate attempt to “fix” underperformance (Tight, 2023). Zabrodska et al. (2011), p. 717) similarly observe that such behavior is “co-implicated in, and justified by, the alleged need for control and improvement of performance.” In academia, the lack of in-depth trauma-informed training is particularly concerning. Academic bullying can take many forms, ranging from psychological harassment to acts of psychological violence. This commonly involves gaslighting, ostracism, public humiliation, and professional sabotage. These behaviors and experiences can deeply impact targets, leading to emotional distress, anxiety, depression, suicidal ideation, and, in some cases, lasting mental health diagnoses such as PTSD. Beyond the psychological effects, physical health issues can also arise, and in more extreme cases, unaddressed academic bullying may result in suicides (Khoo, 2010; Conway et al., 2022; Luo et al., 2023). Yet without trauma-informed education, bystanders and leaders frequently underestimate the severity of the harm inflicted, minimize the experiences of targets, and mismanage interventions or complaint processes, often resulting in further harm or re-traumatization.

The Canadian Institute of Workplace Bullying Resources (Canadian Institute of Workplace Bullying Resources [CIWBR], 2024) highlights a major shortcoming in current training programs. They stress that academic bullying should not be treated merely as a matter of policy enforcement but as a profound human rights issue that requires empathy, understanding, and skillful handling. The CIWBR advocates for trauma-informed training that educates participants about the neurobiological and psychological effects of prolonged exposure to bullying and harassment, while also providing practical strategies for early intervention, recovery support, and systemic prevention. This training emphasizes the importance of changing how one behaves and makes decisions in everyday interactions to prevent further harm and support healing (Canadian Institute of Workplace Bullying Resources [CIWBR], n.d.). A trauma-informed approach is grounded in six key principles: safety; trustworthiness and transparency; peer support; empowerment, voice, and choice; cultural sensitivity and inclusion; and collaboration.

The CIWBR’s most urgent recommendation is the mandatory implementation of trauma-informed training for all leaders, including senior administrators, department chairs, and supervisors. Leaders play a critical role in setting the tone and shaping institutional culture. Without appropriate and sufficient training, they may unconsciously perpetuate harmful practices, fail to recognize early warning signs often dismissed as mere “personality conflicts,” or mishandle reports of misconduct. Trauma-informed training equips leaders with the tools to respond proactively, reduce stigma, promote psychological safety, and lead with emotional awareness to prevent harm.

Universities and workplaces risk perpetuating harm through dismissive, inadequate, or retraumatizing responses when institutional policies and leadership training lack embedded trauma-informed frameworks. The basic bullying awareness programs offered at many institutions often serve as a barrier to bystander action. Training should exceed simple checklists and policy overviews to be truly effective. A comprehensive and trauma-informed model that focuses on cultivating a culture of empathy, accountability, and psychological safety can reduce bystander inaction. Moreover, the integration of trauma-informed principles into leadership practices is essential for transforming academic and workplace environments into spaces where respect and wellbeing are prioritized for all community members.

### 2.3 Laissez-faire leadership

Laissez-faire leadership is characterized by a hands-off approach, minimal support, and decision-making avoidance (Stone, 2025), which allows for the autonomy desired by independent self-directed, creative researchers. However, the lack of direction can decrease motivation and performance (Chhom et al., 2024), and the lack of accountability allows workplace bullying to flourish (Ågotnes et al., 2021). It is the leader’s responsibility to recognize and address these barriers because, unaddressed, they enable systemic harm. Moreover, those in middle management routinely assume leadership roles without the competence or interest in management skills, increasing the risk of laissez-faire leadership behaviors (Rudhumbu, 2015; Machovcová et al., 2024; Tsuno and Kawakami, 2014). These roles are often temporary and lack substantial formal authority, particularly when it comes to disciplining faculty, so interpersonal problems such as bullying become difficult to address (Keashly and Neuman, 2010). Consequently, institutions frequently respond with inaction or ignorance, creating conditions such as favoritism, resentment, and unresolved grievances that fuel bullying and toxic group dynamics. This passive approach fosters a culture of fear rather than safety, ultimately empowering perpetrators and enabling institutional protection of misconduct (Hodgins et al., 2024).

### 2.4 Lack of accountability

Accountability is a critical element in both workplace and educational settings, and leaders are responsible for establishing and maintaining a culture of psychological safety, respect, equality, diversity, and inclusion (Edmondson, 1999; Universities UK, 2023). Leaders carry a duty of care to protect the psychological wellbeing of staff and students, which requires role-modeling, self-reflection, humility, and a commitment to continuous learning (Fisk and Daoust, 2025). Because leadership behavior sets the tone for institutional culture, visible ethical conduct, empathy, and responsible decision-making are essential for fostering trust and psychological safety (Zhang and Song, 2020; Edmondson, 1999). Faculty leadership has a clear responsibility to ensure accountability not only for themselves but also throughout their departments. This involves advocating for students and staff who may be hesitant or afraid to speak up. Leaders should be equipped through mandatory, trauma-informed training to recognize harmful behaviors, understand their impact, and intervene appropriately. Creating a psychologically safe academic environment requires active leadership that fosters trust, models respectful behavior, and consistently manages and addresses negative behaviors in a fair, transparent, and timely manner. By fulfilling these responsibilities, academic leaders can play a pivotal role in transforming institutional culture, ensuring that psychological safety and wellbeing are prioritized for everyone within the academic community.

### 2.5 Implicit or unconscious bias

Implicit or unconscious bias refers to automatic, deeply ingrained tendencies that influence judgment and decision-making based on underlying assumptions or stereotypes. These biases often lead to harmful stereotyping and flawed decision-making, particularly in areas such as mentorship, collaboration, admissions, and faculty promotions (Allen and Garg, 2016). In academic settings, unconscious bias frequently manifests as microaggressions, subtle, often unintentional behaviors or comments that convey demeaning or dismissive messages to members of marginalized groups (Nadal et al., 2011). Over time, these microaggressions contribute to a hostile and exclusionary environment, a problem that is especially pronounced in STEM fields.

To address these pervasive issues, interventions such as the EQUIP web application have been developed to help educators identify and mitigate bias in real time within educational settings (Reinholz et al., 2020). EQUIP is a free application that helps educators analyze classroom participation by coding and visualizing data from video recordings or live observations. Educators customize demographic characteristics like race and ethnicity, as well as other classroom dimensions they wish to track, which can then be analyzed for patterns in classroom discussion. By making participation trends visible, EQUIP supports informed, targeted changes in teaching practices. Tools like this are a crucial step toward building more equitable and inclusive academic environments.

## 3 Conceptual model: the bystander intervention framework in academic bullying

Latané and Darley’s (1970) seminal Bystander Intervention Model outlines a five- step cognitive process that a bystander completes before intervening in an emergency. First, the individual should notice that something is occurring; if the signs are subtle or if one is distracted, the event may go undetected, with no further action performed. Second, the bystander should interpret the situation as a genuine problem or emergency. Ambiguity, as when others appear calm or when cues are minimal, can lead to misinterpretation (Latané and Darley, 1970; Hortensius and de Gelder, 2018). Third, the individual should assume personal responsibility to act. In group settings, diffusion of responsibility is common; each person thinks that someone else will take charge, thereby reducing the incentive for any one person to intervene (Latané and Darley, 1970). Fourth, the bystander should decide how to help by selecting an intervention strategy (e.g., direct action, calling for help, etc.). Without adequate knowledge or confidence in their ability to help, individuals may hesitate even if they feel responsible. Finally, the chosen action should be implemented. Even after deciding to intervene, last- minute doubts or fears (such as social inhibition or fear of retaliation) may prevent actual intervention (Latané and Darley, 1970; Hortensius and de Gelder, 2018).

Each step in this process presents potential barriers; failure to notice or correctly interpret the event halts the process before responsibility is assumed; diffusion of responsibility may inhibit taking personal accountability; and inadequate skills or fear can obstruct the execution of effective help. This model has been applied to understand the inaction in other contexts, such as school, college bullying (Danielson, 2019) and workplace harassment (Hellemans et al., 2017).

### 3.1 Adaptation for academic bullying

Academic bullying differs from sudden emergencies in that it is typically chronic, subtle, and interwoven with institutional hierarchies (Pyke, 2018). First, recognizing bullying in academia often requires a comprehensive understanding of its definition, including risk factors, warning signs, and common tactics and the ability to identify patterns in what may initially appear as isolated incidents, such as disrespectful remarks, exclusion from meetings, or harsh emails, rather than a single overt act. Without this understanding, many fail to see the damage being done. As the saying goes, it is “death by a thousand cuts” (Nadal et al., 2011), a metaphor for the slow, cumulative harm inflicted through repeated minor slights. While often attributed to ancient Chinese execution methods, the phrase has become widely used to describe psychological harm that accrues over time. Targets and witnesses may endure hundreds of subtle abuses, such as being ignored, mocked, glared at, or excluded, before anyone recognizes that real injuries have occurred. People often feel too embarrassed or unsure about reporting such behavior, fearing they will be dismissed as oversensitive. However, as Einarsen (2005) points out, while individual acts may appear trivial in isolation, their cumulative effect can cause deeper psychological harm than a single violent event.

Second, interpreting these interactions as bullying can be challenging due to ambiguous norms regarding feedback and independence in academic settings; hierarchical and cultural factors may lead bystanders to rationalize harsh behavior as acceptable mentoring rather than abuse. Increasing education and awareness at the institutional level is a crucial first step in addressing academic bullying. Varying perceptions of mistreatment in academic and clinical environments often contribute to underreporting and bystander inaction (Colenbrander et al., 2020; Manuel et al., 2023).

Third, in environments with clear power structures, individuals, whether students, postdoctoral researchers, or junior faculty, might assume that someone in a higher position (e.g., a department chair or ombudsperson) is responsible for intervening, further diffusing personal responsibility (Latané and Darley, 1970).

Fourth, deciding how to help is often stalled by uncertainty about the right action—“knowing how to help” is a distinct step in the Latané and Darley bystander decision model, and subsequent studies show that uncertainty about whether to confront, report, or privately support the target reduces intervention in campus and workplace settings (Latané and Darley, 1970; Bennett et al., 2014; Haynes-Baratz, 2021).

Finally, taking action in academic settings can lead to uncertain outcomes. For example, a formal investigation may expose the bystander to backlash, which can reinforce a culture of silence or inaction. Moreover, the institutional response to an intervention (whether supportive or punitive) creates a feedback loop that shapes future bystander behavior.

**FIGURE 1:**
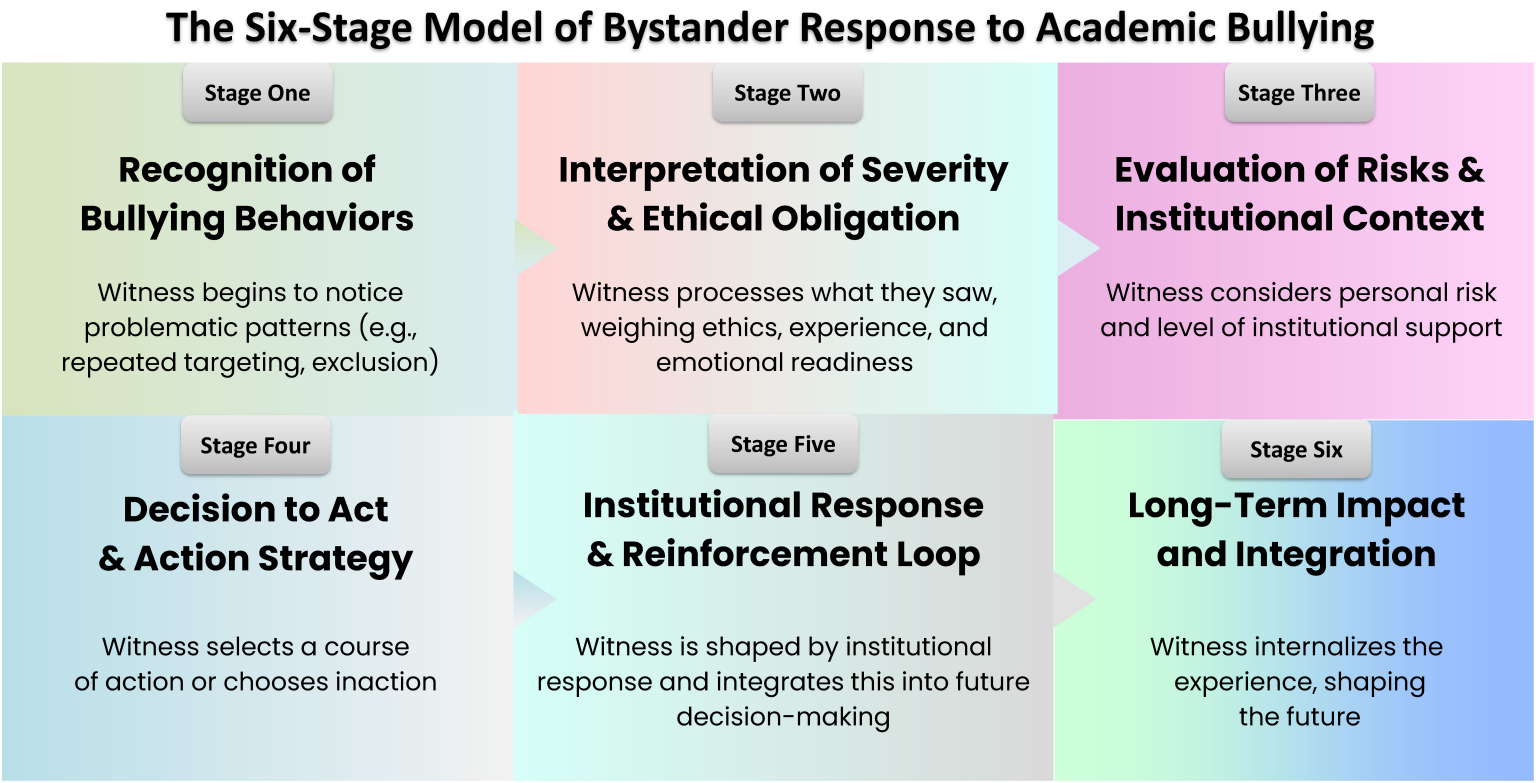
Bystander decision-making model in academic bullying. The flowchart illustrates the iterative process of bystander intervention in academic settings. The model outlines six key stages: recognition of bullying behavior, interpretation of the severity and ethical obligation, evaluation of personal risks and institutional context, the decision to act (with various intervention options), and the subsequent institutional responses (supportive or dismissive), which together form a feedback loop that shapes future intervention behavior.

While Latané and Darley’s (1970) model provides a valuable scaffold (notice → interpret → assume responsibility → decide how to help → act), its application to academic bullying necessitates adjustments to account for the chronic and institutionalized nature of the problem. Academic bystanders should accumulate evidence over time to discern patterns of abuse, overcome normalized cultural cues that obscure the severity of behavior, claim responsibility despite entrenched hierarchies, and navigate complex intervention strategies with long-term consequences. This adapted framework, illustrated in Figure 1, embeds the six- step process within the academic context by highlighting these unique decision points and incorporating an explicit “institutional response and reinforcement” and the sixth “Long-Term Impact and Integration” stage.

### 3.2 Stages 1–3: noticing, interpreting, responsibility (reasons/enablers)

#### 3.2.1 Stage 1: recognition of bullying behavior-noticing the problem

The recognition process begins with one or more bystanders who recognize and label behavior as bullying. In the academic context, this may not be immediate or obvious. Initial signs may be subtle. Consider the following examples: A student is repeatedly interrupted and humiliated in lab meetings by his/her supervisor, or colleagues notice that a senior advisor in faculty discussions consistently dismisses a junior professor’s ideas. On their own, each incident might be written off, but taken together, a pattern emerges. The bystander’s first challenge is to *notice and identify that pattern*. Often, this requires attentiveness and understanding that the behavior is bullying. For instance, a postdoc might begin to realize that during every group meeting, a particular professor harshly criticizes one student more than others. This highlights the importance of attention, without which these behaviors may go unnoticed. Perhaps faculty in a department share notes and collectively discover that this professor has a history of discouraging students with caustic behavior, signaling the need for communication. In many cases, targets of bullying may confide in a trusted peer, helping bystanders understand their observations as part of a larger continuum of mistreatment. However, in academia, the prevalence of territorial behaviors and the “Gollum effect” (Valdez et al., 2025), where researchers hoard resources and obstruct others to maintain a competitive edge, can make it challenging to find supportive colleagues.

One key aspect of recognition is perceiving certain behaviors as *unacceptable*. However, this ability is made more difficult without attention and special instruction. If people receive training to identify bullying patterns and behaviors, they will be more successful in noticing the problem (Manuel et al., 2023). Conflicting perceptions of bullying behavior can lead to underreporting and “bystander silence,” as some witnesses may not classify the behavior as harmful. This issue is compounded in settings where such behaviors are normalized (Neuman and Baron, 2003). For example, if aggressive feedback on grant proposals is considered standard in a lab, members may not interpret it as bullying, even if it escalates into personal attacks. Therefore, developing a shared understanding of bullying, through common definitions and examples, is essential during the initial stage of bystander intervention. Without recognition of bullying behavior, a bystander will not progress to the next steps of intervention. “*It is recommended that a clear definition of academic bullying be explicitly incorporated into institutional policies and equity statements.”* Academic bullying refers to persistent and hostile behavior perpetrated by someone in a position of academic authority. This may include but is not limited to, mockery, threats, unwarranted blame, invasions of privacy, public humiliation, and obstruction of academic or career advancement. Specific tactics can involve withdrawal of funding, unjustifiably negative reference letters, misappropriation of credit for others’ work, and threats to revoke visas or fellowships (Moss and Mahmoudi, 2021). It is important to note that extreme incidents can sometimes prompt bystanders to recognize bullying. For example, a supervisor throwing a notebook at a student in anger is an obvious act of abuse, leading bystanders to conclude, “This is bullying.” However, extreme incidents are infrequent; more often, there is an accumulation of moderate incidents which are hard to label as bullying as academic bullies are often able to “leave no trace” because they tend to be intelligent enough to avoid leaving evidence of their inappropriate actions, for example by using private conversations rather than written or public communications to exert power over targets (Mahmoudi, 2019). Another clear indicator of bullying is the visible distress shown by the target such as social withdrawal, increased isolation, stress-related declines in performance, or emotional dysregulation all of which can signal to attentive bystanders that something is seriously wrong. In sum, Stage 1 of bystander intervention in academia involves an ongoing observational process. Once a potential pattern is noticed, the bystander can interpret it as a form of bullying.

#### 3.2.2 Stage 2: interpretation of severity and ethical obligation

After recognizing bullying behavior, the bystander should interpret it as a problem that requires intervention. This next stage involves judging the severity and unacceptability of actions and feeling a moral responsibility to intervene. In academic settings, several factors influence these interpretations.

##### 3.2.2.1 Ambiguity reduction

Bystanders should reduce the ambiguity of interpretations by assessing the plausibility of alternate explanations. This requires considering the context. Bystanders might use observations regarding consistency, consensus, and distinctiveness (Kelley, 1973) to guide interpretations of bullying behavior. They might ask questions such as: Was the professor’s outburst a one-time thing on a bad day or part of a consistent pattern? Is this an instance of particular student mistreatment or typical constructive academic criticism? Do other professors behave similarly? Bystanders consider both personal beliefs and available institutional guidelines during interpretation processes. However, ambiguity is higher in environments with unclear anti-bullying norms. Universities with official statements condemning intimidating and disparaging behaviors increase bystander confidence levels when labeling these violations. Universities should adopt clear and comprehensive definitions of *academic bullying* within their institutional policies, an area where many currently fall short. In cases where internal action proves insufficient, external mechanisms may be required to ensure accountability and drive meaningful reform. Along with bullying, another pandemic is mobbing, although, beyond the scope of this article, mobbing can be defined as an act when two or more individuals collectively target a person with bullying. This group dynamic amplifies harm, reinforcing shame, isolation, and self-doubt for the target (Leymann, 1996). Similarly, lateral violence refers to harmful behaviors like bullying, gossiping, or sabotage between colleagues at the same hierarchical level. Often stemming from unaddressed systemic stress, competition, or cultural tensions, it is particularly common in high-pressure workplaces (Longo and Sherman, 2007). It is important to recognize that asking questions, seeking clarification, validation, or guidance, particularly to interrupt or report abusive behavior, is not gossip, mutiny, lateral violence, or mobbing. These are responsible actions grounded in due diligence and ethical concern. Martinescu et al. (2019) find that both feedback and gossip provide important evaluative information about an individual’s abilities and behavior, though their intentions and audience differ. Feedback is given to help the recipient, whereas gossip is shared broadly and for various social purposes. This kind of information is especially important in environments where formal guidance is lacking or trust in institutional processes is low.

##### 3.2.2.2 Empathy and moral judgment

The bystander’s level of empathy for the target plays a role in guiding interpretations. A qualitative study conducted by Thornberg et al. (2012) examined how empathy influences helping behavior in school children, finding that emotional reactions are central to the decision to help. Target identification, through friendship or experiences of past discrimination, can increase perceptions of severity and the likelihood of intervention (Thornberg et al., 2012). Empathy strengthens this sense of *ethical obligation*. In academia, a senior professor with a strong moral compass might feel outraged upon witnessing a junior colleague being publicly insulted, increasing the likelihood of perceiving it as an ethical breach that should be addressed. Conversely, if bystanders experience *moral disengagement* by rationalizing the bully’s actions and believing the target is too sensitive and deserving, they may downplay the severity of the issue. When moral disengagement is low, empathy encourages bystanders—students, faculty, staff, and researchers alike to defend victims (Gholiyah et al., 2021). Thus, compassionate, community-oriented institutional cultures boost bystander condemnation and pro-social action, while individualistic, competitive cultures suppress it. An important avenue for future research would be a cross-cultural investigation (collectivist vs. individualistic) of this model to better understand how cultural contexts shape responses to academic bullying.

##### 3.2.2.3 Pluralistic ignorance and social cues

If many individuals witness a professor’s harmful comment and fail to intervene, each person may interpret the lack of response as an indication of low severity. This example illustrates pluralistic ignorance. Pluralistic ignorance occurs when bystanders misinterpret collective inaction as tacit approval (Hortensius and de Gelder, 2018). Deference to authority and group conformity (Asch, 1951) reinforce silence, leading junior members to believe bullying is acceptable. *Status quo* bias (Samuelson and Zeckhauser, 1988) further normalizes hostile behaviors, as newcomers adopt existing norms without question. Yet a single bystander can break this cycle by speaking out and validating others’ concerns (Hortensius and de Gelder, 2018).

##### 3.2.2.4 Ethical/role obligation

Bystander intervention in cases of academic bullying hinges on individuals’ perceptions of responsibility, which are influenced by their departmental rank, personal ethics, and moral courage. Department chairs and senior faculty may either intervene promptly guided by professional codes, or remain silent to protect institutional reputation. Professional ethics statements (e.g., American Association of University Professors [AAUP], 2007) underscore respect and non-harassment among faculty, and many universities now embed “bystander responsibility” clauses in their conduct policies (Vaill et al., 2023). Empirical evidence suggests that well-publicized anti-bullying policies can reduce incidents (Nikolaou, 2017), yet students and faculty often lack awareness of these guidelines (Vaill et al., 2023; Lund and Ross, 2017). To foster effective bystander action, institutions should clearly communicate ethical expectations and policy details to the entire academic community.

By the end of Stage 2, the ideal outcome is for the bystander to recognize bullying as *wrong, harmful*, and in need of *intervention*. It is only after this process that bystanders can take action. However, if the bystander(s) hesitate or dismiss the bullying as normal, insignificant, or the target’s fault, they are less likely to complete this interpretation and may remain passive bystanders. Education and awareness initiatives can enhance this stage by providing clear examples of bullying and reinforcing its recognition as an ethical violation (Manuel et al., 2023). When people are equipped with knowledge and clarity, they are more likely to shift from passive observation to active disruption, even in complex or high-risk academic environments.

#### 3.2.3 Stage 3: evaluation of risks and institutional context

If bystanders recognize bullying incidents as serious and needing assistance, they should consider the practical aspects of the situation. This involves self-reflection on risks and available support. *What are the risks I face if I help? What support can I expect from my institution? Will I face reprisal?* In Stage 3, pro-sociality frequently intersects with self-preservation and practical considerations, especially in academia. The following factors should be considered.

##### 3.2.3.1 Fear of personal repercussions

In this stage, the bystander starts to evaluate possible repercussions, with fear of consequences playing a prominent role. Some valid questions arise: *If I intervene, will I be targeted too? Could I lose my job, funding, or connections?* Risks may appear more significant for a junior-level colleague with less institutional experience and protection. Even senior members may have underlying concerns about creating conflicts and harming collegial relationships. Moreover, there is a risk of social exclusion, particularly if the bully has powerful allies. These fears are real, as surveys indicate that many academics feel reporting misconduct can lead to negative consequences for the individual reporting it (Proudhon, 2025; Jönsson and Muhonen, 2022). Prior work reports a non-trivial risk of reprisals following challenges to misconduct (Täuber et al., 2022). Faculty members may recall how colleagues’ perceptions shifted negatively after someone filed a complaint. As a result, they may fear becoming targets themselves or facing social and professional consequences, such as reduced opportunities for collaboration or career advancement (Jönsson and Muhonen, 2022). These situations often involve cost–benefit analyses, where ethical obligations are weighed against personal risk. Unfortunately, inaction tends to prevail unless moral conviction or identification with the target is particularly strong.

##### 3.2.3.2 Power dynamics and status

In academia, power dynamics play a crucial role in determining whether bystanders help (Corbett et al., 2024). Their status influences both their perceived risk and ability to intervene. As previously mentioned, students and postdocs, non-tenured faculty staff, and even some administrators hold less power and face greater risks compared to tenured professors, who have more job security and social capital to denounce bullying behaviors. Even pre-tenured professors may hesitate to take action due to fewer benefits and greater uncertainty. Additionally, bystanders from marginalized groups, such as women in male-dominated environments or international scholars in unfamiliar systems, are more vulnerable. A qualitative study conducted by Jönsson and Muhonen (2022) found that passive behavior among bystanders was more common among individuals outside the dominant group. For instance, international students might worry that challenging an American professor could jeopardize their visa status or negatively impact their long-term prospects. Further, intersectional identities, where individuals belong to multiple marginalized groups, create additional barriers to offering help and heighten perceived risk. Ultimately, status and identity shape how bystanders evaluate risk and perceive support within their institution.

##### 3.2.3.3 Institutional history and climate

Another factor the bystander will consider is their institution’s history in responding to bullying incidents, which may have been handled effectively or poorly. Knowledge of successful instances where bystander reports were addressed and disciplinary measures taken can encourage bystanders to act (these success stories are rare and are not often available to bystanders). It is important to know that “my university will support me, and something will be done.” However, this is less likely than a target being aware of widespread institutional inaction. Many targets report ineffective responses, reputation damage, ruined careers, and being forced to continue working with a bully (Moss and Mahmoudi, 2021). This reinforces the perception that institutions tolerate bullying and may not support those who come forward (Jönsson and Muhonen, 2022; Tsuno and Kawakami, 2014). Therefore, the perception of the educational climate is crucial. With low perceived support, bystanders may rationalize not raising concerns if they believe nothing will come of it.

In contrast, an administrator who explicitly condemns bullying and urges community members to speak out fosters a climate of high perceived support. Universities that denounce bullying behaviors and are committed to enforcing anti-bullying measures create an environment where bystanders feel comfortable speaking out. Their fears may be alleviated if, for example, they recall the President’s assurance that retaliation against those who speak up will not be tolerated. Transformational leadership, modeling ethical behavior, inspiring action, and addressing individual needs, has been associated with climates perceived as more supportive of speaking up (Bass and Avolio, 1994). Whereas a lack of transparency regarding anti-bullying actions is prevalent across academic settings (Proudhon, 2025), impacting bystanders’ perceptions of institutional history and climate. Often, the average bystander ends up believing that their risk is too high and institutional support is too low.

##### 3.2.3.4 Options and efficacy

This stage also involves considering specific ways to help and evaluating the feasibility of each option. The bystander may ask, *“What paths are available to me?”* They might choose to reach out to the target, report the issue to a senior colleague, file a formal complaint, or take another course of action. However, each option comes with its own risks. If available, anonymous reporting offers the lowest personal risk compared to more direct or confrontational methods. The option to message a hotline or meet with an ombudsperson can encourage bystanders to take action by providing a safer alternative. The bystander should ask, *“How can I effectively intervene?”* The answer lies in whether they feel prepared to engage. This includes knowing who to speak to, understanding institutional roles (e.g., ombudsperson, HR, union rep), having a basic sense of what the process may entail, and how to initiate contact with someone in authority. Confidence increases when there is clarity: *What steps should I take? What will happen after I report? Who will follow up?* Knowing these details helps reduce hesitation and fear of escalation. These uncertainties contribute to a perceived risk of failure, making self-efficacy a key factor. Specialized training and awareness may bolster a bystander’s confidence in selecting the best intervention strategy. Knowing the 5 Ds (Direct, Distract, Delegate, Delay, and Document) of bystander intervention and rehearsing how to approach reporting can reduce barriers to helping and reporting (Right To Be, n.d.). If internal mechanisms prove ineffective or unsafe, bystanders are strongly encouraged to seek external support services for professional guidance on how to proceed. Conversely, a lack of preparation may increase psychological risk, leading to anxious thoughts and hyper-focusing on negative outcomes.

Overall, Stage 3 is a critical decision-making period. At this stage, the witness feels compelled to act but begins to grapple with questions about potential risks and the best course of action. Building on the previous stage, the bystander has the drive to intervene, but questions about risks and how to proceed emerge. They should internally process potential outcomes and assess their level of courage. If the perceived relational and educational costs outweigh their ethical obligations, they may choose not to intervene. In such cases, interventions fade from the cost-benefit analysis, which favors self-interest. However, if the situation is severe, some bystanders cannot morally dismiss it; if they see a safe path forward, they will move to the next stage. These individuals act heroically, accepting personal risks to defend what they believe is ethical.

### 3.3 Stages 4–6: options, action, institutional response (interventions)

#### 3.3.1 Stage 4: decision to act (or not) and action strategy

This stage builds on the previous ones: recognizing bullying, feeling ethically obligated, and deciding that action outweighs the risks. Here, the bystander makes a concrete choice. If they act, they should form a plan: *What steps should I take? When? What happens if I do nothing?* A bystander may choose one or more of the following strategies if they are appropriate and achievable:

##### 3.3.1.1 Direct intervention

This strategy involves addressing the bully’s behavior either during or shortly after the incident. In academic settings, speaking out in the moment can be especially effective (Latané and Darley, 1970; Banyard et al., 2004). For example, if a bystander overhears a student being disparaged, they might call out the professor’s inappropriate tone or redirect the conversation away from the target. Offering the target affirming comments or shifting attention can help de-escalate the situation and reinforce social support (Banyard et al., 2004). Intervention can also occur immediately after the event by privately confronting the aggressor; however, this method is riskier and is generally more feasible for colleagues with high confidence and status than for subordinates, who may be more easily dismissed or threatened (Einarsen et al., 2011). Though student or junior-colleague intervention remains valuable, they can often adopt subtler tactics like expressing disapproval non-verbally (e.g., refraining from laughing at insulting jokes) or asking the professor to rephrase a comment if it seemed rude, which are instrumental in signaling norms without escalating risk (Latané and Darley, 1970). In addition, private conversations with the aggressor that use clear, specific examples of unacceptable actions, framed professionally and with reference to institutional policies, can encourage accountability and reduce repeat offense (Einarsen et al., 2011). Similarly, speaking privately with the target to validate the harm they experienced, affirm that the behavior was undeserved, and remind them of available policies, procedures, and resources reinforces support and empowers them to take further steps if desired. Even passive resistance can be risky in academic environments where inflated egos or narcissistic traits are present. Online anonymity and disinhibition can further amplify harassment, so caution and strategy are required (Suler, 2004). This underscores the urgent need for comprehensive prevention and intervention training, which equips individuals with the communication tools and protective strategies necessary to navigate these dynamics safely, ethically, and effectively (Banyard et al., 2004; Einarsen et al., 2011).

##### 3.3.1.2 Indirect intervention via authority (reporting)

One strategy is to report to an authority with the status and influence to appropriately handle the issue, as only certain actors can implement change (Gewin, 2021). Bystanders may choose to consult the department chair, a graduate program director, or the university ombudsperson, depending on the nature of the transgression. Additionally, formal procedures or special governing bodies can play a role in intervention (Gewin, 2021). For instance, there may be a dedicated office for specific types of misconduct, such as Title IX for sex-based discrimination or a harassment officer. If, for example, university policy states that bullying complaints should be directed to the Office of Equal Opportunity, this makes the choice easier. In the absence of formal pathways or if they are not well-advertised, a bystander is more likely to seek guidance from a trusted colleague. These decisions are critical in determining whether action is taken. Reporting can be done confidentially or on the record. Anonymous reporting, through a hotline or other online channels, can be a safer option for mitigating personal and professional risk. However, anonymous reports may only lead to university-level preventive measures rather than individual consequences (Gewin, 2021). Individuals with firsthand experiences of bullying in academia were consulted by the authors, and it was emphasized that all key roles ultimately report to the central administration; if wrongdoing is rooted at that level, the entire response process can be compromised. In academia, targets are often hesitant to report, making bystander intervention one of the most effective and influential strategies (Salmivalli, 2010). Some bystanders may serve as witnesses in formal complaints. When multiple bystanders come forward, there is safety in numbers. For example, a group of postdocs collectively taking action against a lab leader carries significant weight, as it lends greater credibility and reduces individual risk.

##### 3.3.1.3 Support the target (comfort, counsel, empower)

Not every action strategy requires confrontation or reporting. Sometimes, the best support comes from simply being there for the target. Ulz (2022) emphasizes the importance of supporting colleagues who experience bullying. A bystander can offer support by privately checking in on the target’s emotional wellbeing, providing advice, and reassuring them that the incident was not their fault. This social support mediates the mental health effects of victimization (Lin et al., 2020), easing the target’s sense of isolation and validating their experience. This is especially crucial in environments where group silence is common.

Bystanders also need dedicated support; they require a psychologically safe space where they can process their own fears and dilemmas around reporting, receive validation for their emotional responses, and explore strategies that align with their personal safety and the unique power dynamics of academic environments. Research has shown that bystander intervention is influenced by complex factors such as role ambiguity, organizational hierarchy, and situational context, particularly within universities and similar institutions (Scully and Rowe, 2009).

Additionally, in workplace settings, coworker support can buffer against bullying; however, this effect is small and less protective for women and persons of color (Attell et al., 2017). Research shows that targets with at least one defender report higher self-esteem than those without supporters, who are often less accepted and more frequently bullied (Sainio et al., 2010). A supportive bystander might also encourage the target to seek professional help or report the incident (Ulz, 2022), such as a graduate student offering to be a witness and corroborate their story. Unions in academic settings may serve as upstanders to support the target as well as other bystanders.

##### 3.3.1.4 Document and gather evidence

Bystanders can document bullying or encourage colleagues to track harmful behaviors and comments (Ulz, 2022). For instance, lab members may quietly document an instructor’s harassment. Dates and details can strengthen formal actions. While this does not stop the bullying immediately, it can help prevent future targeting by providing evidence when reporting. Documentation is a key part of the 5Ds *(Right To Be, n.d.)* taught in harassment training. These small steps carry little risk for bystanders who are not ready to file formal complaints but still make a difference, especially during investigations. Moreover, the inherently territorial nature of academia (Valdez et al., 2025) significantly reduces the likelihood that bystanders will intervene or speak up unless institutions actively promote awareness and implement mandatory reporting mechanisms.

**FIGURE 2:**
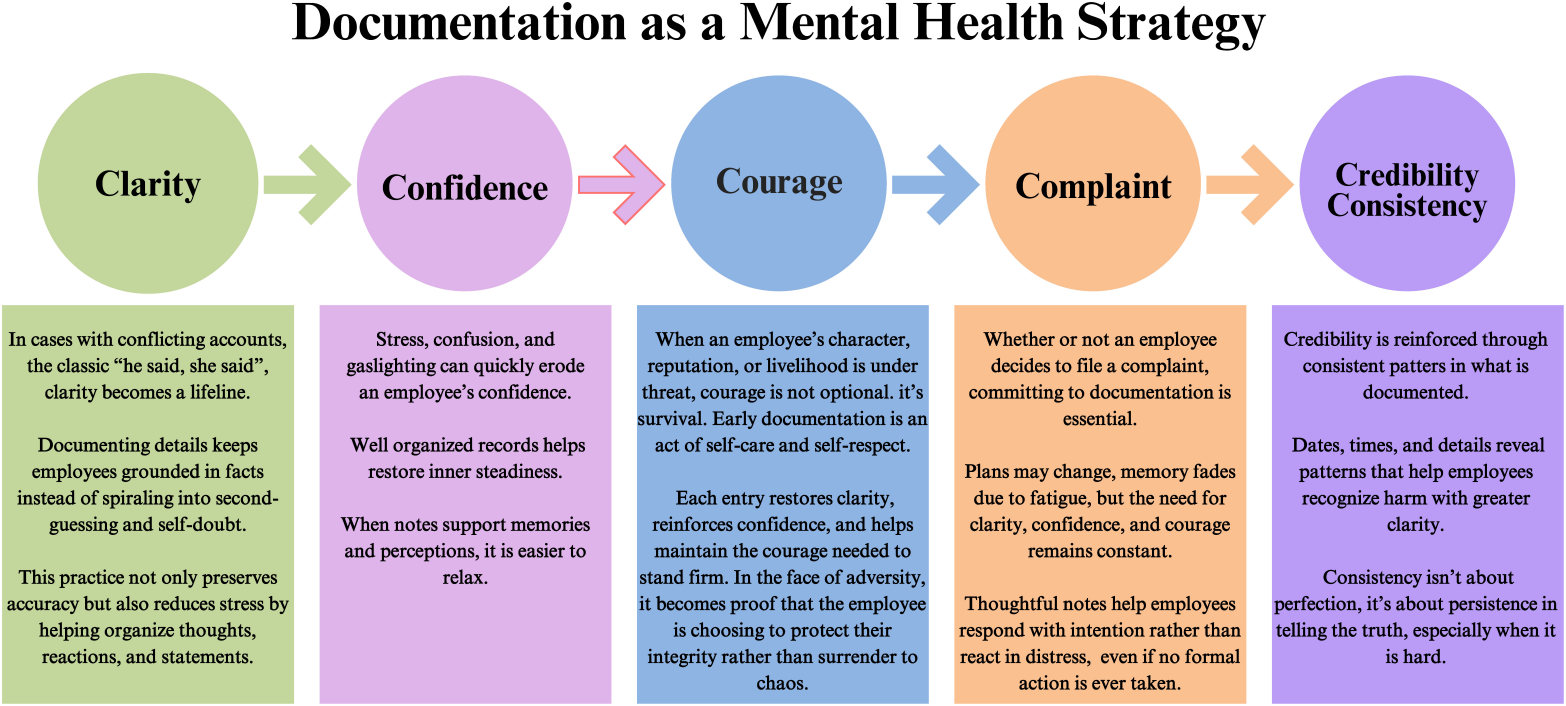
Documentation as a mental health strategy: a five-step framework of clarity, confidence, courage, complaint, credibility, and consistency, illustrating how detailed record-keeping helps employees stay grounded, rebuild self-trust, act decisively, respond thoughtfully, and reinforce their integrity when facing workplace adversity. This figure is adapted from an unpublished original work by co-author Linda Crockett, used with permission.

Witnessing or experiencing academic bullying is mentally and emotionally fatiguing, and many important details are often forgotten over time. Documentation is not just about building a case; it is also a vital act of self-preservation and mental health management. As outlined in Figure 2: *Crockett’s 5Cs of Documentation*, when we are stressed and fatigued, our clarity is the first thing to go. If clarity depletes, so does our confidence. Without clarity and confidence, our courage weakens, and we need all three to come forward when the time is right. Maintaining a consistent record helps preserve clarity, rebuild confidence, and ultimately supports the courage required to file a formal complaint. Consistent documentation ensures not only the strength of the case but also reinforces the credibility of the witness. From a mental health perspective, the act of documenting gives the witness a sense of control, reduces rumination, validates their experience, and creates emotional distance from the chaos all of which are essential for psychological recovery and stability.

When bystanders fail to act, they remain passive observers and become complicit. Their silence, whether through laughter or a failure to challenge exclusionary behaviors, can subtly condone the bully’s actions (Song and Oh, 2018). The bystander may rationalize their inaction by claiming that confrontation is too costly and not their responsibility, or that they will intervene if the situation worsens. This rationale reduces internal moral conflict while also indefinitely deferring action.

Some bystanders may seek *informal resolutions* through their “actions.” For example, a faculty member might privately address the bully, prompting them to reconsider their behavior. If the bully was unaware of their insensitivity, they may be open to change, especially if they recognize the severity of their actions. This approach helps avoid a larger confrontation. Anyone who chooses to seek informal resolutions by directly addressing the person causing harm should always bring concrete examples, including dates, times, what was said, how others reacted, and the impact on themselves or the environment. This level of clarity supports a confident and authentic tone that is direct and outcome focused. At the end of Stage 4, the bystander either acts or deliberately chooses inaction. If they choose to act, they should decide which of the approaches to take.

#### 3.3.2 Stage 5: institutional response and reinforcement loop

After the bystander selects their approach and implements their action plan, institutional responses influence how the current outcomes will affect future ones. This stage extends Latané and Darley’s (1970) original model to capture the iterative process of bystander bullying intervention in academic institutions. Two broad scenarios are presented:

##### 3.3.2.1 Positive/effective institutional response

At this point, the bystander has taken action, or the target has been encouraged to do the same. The institution, then, plays a role in effectively addressing the issues if they are reported through affiliated channels. Ideally, institutions should respond by conducting a trauma-informed investigation, protecting the complainants, and administering justice after confirmation. This may include mandatory trauma-informed leadership and communication training, anger management or anti-bullying programs, specialized coaching or counseling focused on behavioral change, disciplinary action, demotion from leadership roles, or the implementation of new preventative policies. Universities validate the bystander’s choice to intervene by responding in one or all of these ways. Even informal responses, such as a department chair privately admonishing a bully’s behavior, can effectively signal that the institution will not overlook bullying. Witnessing accountability and institutional support for the target reinforces a bystander’s decision to act. In this context, the concept of *institutional courage* becomes relevant. When wrongdoing is reported, active organizational support and a commitment to addressing issues foster a culture of honesty and safety. An administrator publicly and privately thanking bystanders for speaking up and promptly addressing the bullying is an example of institutional courage. This support empowers bystanders and targets, enabling future intervention. First-hand experience of a positive institutional response will increase the likelihood that bystanders and even their colleagues intervene in the future. Thus, over time, more people will stand up against bullying, it will be addressed more quickly, and it will become evident that the institution and community do not tolerate any form of bullying. As shown in our model (Figure 1), this is a “reinforcement loop,” where positive institutional responses influence the recognition of bullying behaviors and lead to quicker action in the future.

##### 3.3.2.2 Negative/ineffective institutional response

Unfortunately, negative and ineffective responses are common. Survey data reveal that two-thirds of bullying targets attempt to take action but are dissatisfied with the outcomes (Quine, 1999). Direct confrontation can cause retaliation by the bully or escalation of bullying behavior. Additionally, formal reporting can be met with institutional apathy or resistance, including cover-ups and denial of evidence, especially when the bullying is verbal and undocumented. Even when action is taken, responses may be ineffectual, maintaining power dynamics and allowing subtle retaliatory behavior, such as social exclusion or negative performance reviews (Moss and Mahmoudi, 2021). In academia, as in other fields, whistleblowers may risk damaging their careers (Pyke, 2018). For example, a junior colleague who reports a celebrated scientist may find their contract not renewed, advancement restricted, letters of recommendation withheld, and more. These negative outcomes signal to bystanders and the broader community that intervening carries greater risks than staying silent, leading to institutional betrayal where organizations fail to protect their members. Sometimes, individuals who engage in bullying may use similar strategies to elevate favored colleagues to higher positions, thereby dismissing or ignoring complaints of bullying against them (Täuber and Mahmoudi, 2022). The bystander likely experiences psychological distress after intervening, reinforcing the belief, “I can’t do that again; nothing good will come of it.” In these cases, the institutional response serves as negative reinforcement, reducing the likelihood of future intervention. This creates a *chilling effect* when an institution tolerates bullying and punishes those who report it; individuals perceive greater risks in speaking up and view inaction as the norm (Jönsson and Muhonen, 2022; Tsuno and Kawakami, 2014). This reinforcement loop encourages passive behavior, leading bystanders to believe that silence is safer, thereby perpetuating the cycle of silence around bullying. Worse still, bullies may continue their harmful behavior due to the lack of effective consequences (Tsuno and Kawakami, 2014).

### 3.3.3 Stage 6: long-term impact and integration

Ineffective institutional action can be discouraging, even when bystanders face no consequences. A central, futile question of “Why bother next time?” often arises when bullying is not addressed, especially when bystanders have reported through the appropriate channels. A critical component of our model is the feedback effect on future behaviors. Academic bullying is not limited to isolated incidents; it both reflects and shapes broader social contexts and institutional cultures. The effectiveness of interventions directly impacts the likelihood of bullying and future reporting. For example, if a department chair dismisses a junior professor’s concerns about bullying during a faculty meeting, other junior colleagues may hesitate to speak out in the future. Conversely, in departments where bullies are removed from their positions after the dean is informed, bystander intervention is more likely to increase due to a stronger perception of institutional support. It is also acknowledged that false accusations of bullying are possible; however, such instances are presumably unlikely among vulnerable groups, such as international students, who have often invested considerable effort and resources to attain their positions and may tolerate mistreatment until it becomes unbearable. Therefore, it is essential that institutions include a clear and comprehensive definition of academic bullying in their official policies. Furthermore, institutions should empower and equip their staff with proper training and certification to handle such sensitive issues effectively. For example, individuals who are not trained in recognizing and addressing racism should not be entrusted with investigating related complaints, as a lack of expertise can lead to inadequate or biased responses.

Institutional responses shape the long-term impact of the experience and influence how bystanders continue to recognize, assess, and respond to bullying (as outlined in the model). Effective responses can break the cycle of bullying, while inaction allows it to persist. If the bully is stopped, the issue is resolved in that instance, helping to prevent future incidents. However, if no meaningful action is taken, bullying continues, and bystanders may remain passive, reinforcing a cycle of inaction. Institutional responses also shape the target’s experience. Successful intervention fosters a safer environment for healing and growth, whereas ineffective responses often drive targets to leave the institution. Witnessing these consequences, bystanders may hesitate to intervene if they experience bullying themselves. Our framework emphasizes that empowering bystanders requires more than individual courage; it depends on a system that supports, rewards, or at least meaningfully responds to their interventions. When institutions reduce barriers and improve their responses, bystanders are more likely to see action as the better choice in their *cost-benefit analysis* of Stage 3.

To further illustrate the model, imagine the case of “John.” He is a postdoc whose supervisor consistently undermines his intelligence in the lab. His lab mates notice this behavioral pattern (Stage 1) and identify it as severe, especially after witnessing John exhibit adverse psychological effects (Stage 2). These bystanders feel empathy and recognize the supervisor’s wrongdoing. One of them, Jane, now feels obligated to act. She recalls that the university has always emphasized student wellbeing, so she weighs the potential outcomes (Stage 3). Confronting their well-respected supervisor could have repercussions, especially since she is less experienced; thus, anonymous reporting to the department chair feels safer. She emails the chair, describing what she has witnessed (Stage 4 action). An effective response would involve meeting with the supervisor to issue a warning and providing John and others affected with support, such as facilitating a transition to a different mentor (Stage 5 positive outcome). In this scenario, Jane sees that her intervention has positively affected John’s mental health and has not harmed either of their careers. This success motivates her and the rest of the lab to act in future similar situations. However, had the chair ignored the email or conveyed its message to the supervisor, who then retaliated by removing Jane from their project (Stage 5 negative outcome), this would have discouraged her and her colleagues from intervening in the future. Ultimately, this reinforces group silence and emboldens the bully.

This example illustrates how each phase of the model builds on the previous one and highlights the importance of bystander empowerment at every stage. If any step is disrupted whether due to a failure to recognize bullying, fear of retaliation, or a history of institutional inaction, the intervention process collapses. Our model identifies critical areas for strengthening bystander intervention: raising awareness in the early stages (Stage 1–2), fostering supportive communities and clarifying responsibility in the decision-making phase (Stage 3), equipping individuals with safe intervention strategies (Stage 4), and ensuring institutional accountability and protection against retaliation (Stage 5). In the following sections, The barriers that hinder these stages (section 4) are also examined and propose strategies to overcome them (section 6).

## 4 Limitations and future directions

This conceptual model advances theory: its predictive accuracy and ecological validity now invite empirical examination by future researchers. Universities also vary by discipline, rank and culture. How a medicine department works is different from a humanities department. In higher education, prevalence and power asymmetries shift with local cultures and institutional logics (Tight, 2023). Without comparative fieldwork, it is unclear whether the proposed stages will operate similarly for graduate students, junior faculty, and administrators. Research indicates that bystander motivations and norms of intervention vary considerably between individualist and collectivist cultures (Pozzoli et al., 2012). Models developed primarily from Western studies may overemphasize individual agency and overlook important cultural factors such as face-saving and respect for hierarchy that shape bystander behavior in many East- and South-Asian academic environments. The proposed trauma-informed training can be tested in future studies or surveys to assess gains in trauma literacy, bystander self-efficacy, and actual intervention behavior, ideally via intervention trials, such as multi-site randomized controlled trials (RCTs) of trauma-informed and ally-skills programs that oversee both positive action (e.g., increased reporting, peer support) and potential misuse, including false-allegation rates.

## 5 Conclusion

The promise of a safer, more inclusive academia depends on activating both moral conviction and institutional accountability. By reconceptualizing bystanders not as passive observers but as pivotal agents of change, this paper’s six-stage bystander intervention model demonstrates how awareness, empathy, and responsibility can be translated into meaningful action, provided institutions commit to credible, consistent support. When universities embed trauma-informed training, implement transparent reporting mechanisms, and enforce zero-tolerance policies for bullying and harassment, they fundamentally shift the decision-making landscape for bystanders. Risk is no longer borne solely by the individual; responsibility becomes collective. In doing so, institutions can replace cycles of silence with reinforcement loops of active intervention, validation, and systemic support. This convergence of psychological insight and structural reform not only safeguards individual courage but also cultivates academic environments in which targets are protected, bystanders are empowered, and abusive hierarchies are dismantled. Environments that uphold both scholarly rigor and human dignity are only possible when institutions acknowledge that the silence of bystanders is not apathy; it is a rational, learned response to institutional failure. The path forward is clear: empower the witness, and the academic community transitions from passive bystanding to active safeguarding.

## Data Availability

The original contributions presented in the study are included in the article/supplementary material, further inquiries can be directed to the corresponding author.

## References

[B1] Abbott A. (2020). Stress, anxiety, harassment: Huge survey reveals pressures of scientists’ working lives. *Nature* 577 460–461. 10.1038/d41586-020-00101-9 31965094

[B2] Adams-Clark A. A. Harsey S. Freyd J. J. (2024). Factors of institutional betrayal associated with PTSD symptoms and barriers to service use among campus sexual assault survivors. *Psychol. Injury Law* 17 383–397. 10.1007/s12207-024-09524-5

[B3] Ågotnes K. W. Skogstad A. Hetland J. Olsen O. K. Espevik R. Bakker A. B. (2021). Daily work pressure and exposure to bullying-related negative acts: The role of daily transformational and laissez-faire leadership. *Eur. Manag. J.* 39 423–433. 10.1016/j.emj.2020.09.011

[B4] Allen B. J. Garg K. (2016). Diversity matters in academic radiology: Acknowledging and addressing unconscious bias. *J. Am. Coll. Radiol.* 13 1426–1432. 10.1016/j.jacr.2016.08.016 27916109

[B5] American Association of University Professors [AAUP] (2007). *Statement on professional ethics.* Washington, DC: AAUP.

[B6] Asch S. E. (1951). “Effects of group pressure upon the modification and distortion of judgments,” in *Groups, leadership, and men: Research in human relations*, ed. Guetzkow H. (Carnegie Press), 177–190.

[B7] Attell B. K. Brown K. K. Treiber L. A. (2017). Workplace bullying, perceived job stressors, and psychological distress: Gender and race differences in the stress process. *Soc. Sci. Res.* 65 210–221. 10.1016/j.ssresearch.2017.02.001 28599773

[B8] Banyard V. L. Plante E. G. Moynihan M. M. (2004). Bystander education: Bringing a broader community perspective to sexual violence prevention. *J. Commun. Psychol.* 32 61–79. 10.1002/jcop.10078

[B9] Bass B. M. Avolio B. J. (eds) (1994). *Improving organizational effectiveness through transformational leadership.* London: Sage.

[B10] Bennett S. Banyard V. L. Garnhart L. (2014). To act or not to act, that is the question? Barriers and facilitators of bystander intervention. *J. Interpersonal Viol.* 29 476–496. 10.1177/0886260513505210 24097909

[B11] Bloeser K. McAdams M. McCarron K. K. Varon S. Pickett L. Johnson I. (2023). Institutional courage in healthcare: An improvement project exploring the perspectives of veterans exposed to airborne hazards. *Behav. Sci.* 13:423. 10.3390/bs13050423 37232660 PMC10215171

[B12] Branch S. Ramsay S. Barker M. (2013). Workplace bullying, mobbing and general harassment: A review. *Int. J. Manag. Rev.* 15 280–299. 10.1111/j.1468-2370.2012.00339.x

[B13] Brüggemann A. J. Forsberg C. Colnerud G. Wijma B. Thornberg R. (2018). Bystander passivity in health care and school settings: Moral disengagement, moral distress, and opportunities for moral education. *J. Moral Educ.* 48 199–213. 10.1080/03057240.2018.1471391

[B14] Bunce A. Hashemi L. Clark C. Stansfeld S. Myers C. A. McManus S. (2024). Prevalence and nature of workplace bullying and harassment and associations with mental health conditions in England: a cross-sectional probability sample survey. *BMC Public Health* 24:1147. 10.1186/s12889-024-18614-7 38658961 PMC11044501

[B15] Canadian Institute of Workplace Bullying Resources [CIWBR] (n.d.). *Protecting mental health in the workplace.* Available online at: https://instituteofworkplacebullyingresources.ca/protecting-mental-health-in-the-workplace-2/ (accessed June 6, 2025).

[B16] Canadian Institute of Workplace Bullying Resources [CIWBR]. (2024). Available online at: https://instituteofworkplacebullyingresources.ca/ (accessed October 23, 2024).

[B17] Carney J. V. Hazler R. J. (2011). Exposure to school bullying and the social capital of sixth-grade students. *J. Humanistic Counseling* 50 238–253. 10.1002/j.2161-1939.2011.tb00122.x

[B18] Cheng W. Philpot R. Levine M. (2025). The death of ‘Little Yue Yue’—An analysis of public commentaries on an iconic case of bystander apathy in China. *J. Commun. Appl. Soc. Psychol.* 35:e70139. 10.1002/casp.70139

[B19] Chhom C. Vy S. Chheav R. Bou D. Kheuy S. Sam R. (2024). The laissez-faire leadership style in higher education institutions: A systematic literature review. *Eur. J. Contemp. Educ. E-Learn.* 2 140–168. 10.59324/ejceel.2024.2(6).09

[B20] Colenbrander L. Causer L. Haire B. (2020). ‘If you can’t make it, you’re not tough enough to do medicine’: A qualitative study of Sydney-based medical students’ experiences of bullying and harassment in clinical settings. *BMC Med. Educ.* 20:86. 10.1186/s12909-020-02001-y 32209074 PMC7092452

[B21] Conway P. M. Erlangsen A. Grynderup M. B. Clausen T. Rugulies R. Bjorner J. B. (2022). Workplace bullying and risk of suicide and suicide attempts: A register-based prospective cohort study of 98 330 participants in Denmark. *Scand. J. Work Environ. Health* 48 425–434. 10.5271/sjweh.4034 35648097 PMC9888442

[B22] Corbett E. Barnett J. Yeomans L. Blackwood L. (2024). “That’s just the way it is”: Bullying and harassment in STEM academia. *Int. J. Stem Educ.* 11:27. 10.1186/s40594-024-00486-3

[B23] Crusto C. A. Hooper L. M. Arora I. S. (2024). Preventing sexual harassment in higher education: A framework for prevention science program development. *J. Prev.* 45 501–520. 10.1007/s10935-024-00780-4 38613725 PMC11271342

[B24] Danielson C. (2019). *Bystanders to college bullying: An application of the bystander intervention model.* Doctoral dissertation. Minneapolis: University of Minnesota.

[B25] Darley J. M. Latané B. (1968). Bystander intervention in emergencies: Diffusion of responsibility. *J. Personal. Soc. Psychol.* 8 377–383. 10.1037/h0025589 5645600

[B26] Edmondson A. C. (1999). Psychological safety and learning behavior in work teams. *Admin. Sci. Quar.* 44 350–383. 10.2307/2666999

[B27] Einarsen S. (2005). *The nature, causes and consequences of bullying at work: The Norwegian experience. Perspectives interdisciplinaires sur le travail et la santé*. Institut de Recherche Robert-Sauvé en Santé et en Sécurité du Travail (IRSST). 10.4000/pistes.3156

[B28] Einarsen S. Hoel H. Zapf D. Cooper C. L. (eds) (2011). *Bullying and harassment in the workplace: Developments in theory, research, and practice*, 2nd Edn. Boca Raton, FL: CRC Press.

[B29] Escartín J. (2016). Insights into workplace bullying: Psychosocial drivers and effective interventions. *Psychol. Res. Behav. Manag.* 9 157–169. 10.2147/PRBM.S91211 27382343 PMC4924877

[B30] Fisk G. M. Daoust L. E. (2025). Advancing a trauma-informed approach to leadership in the workplace: A conceptual review and theoretical extension. *Psychol. Leaders Leadersh.* 28 131–156. 10.1037/mgr0000172

[B31] Freyd J. J. (1996). *Betrayal trauma: The logic of forgetting childhood abuse.* Cambridge, MA: Harvard University Press.

[B32] Freyd J. J. (2023). *What is a betrayal trauma? What is betrayal trauma theory?.* Oregon: Freyd Dynamics Lab.

[B33] Garland T. S. Policastro C. Richards T. N. Miller K. S. (2016). Blaming the victim: University student attitudes toward bullying. *J. Aggress. Maltreatment Trauma* 26 69–87. 10.1080/10926771.2016.1194940

[B34] Gewin V. (2021). How to blow the whistle on an academic bully. *Nature* 593 299–301. 10.1038/d41586-021-01252-z 33976423

[B35] Gholiyah Y. D. Nashori H. F. Diana R. R. (2021). The effect of empathy to bystander’s role towards bullying at school through moral disengagement as a mediator. *Commun. Human. Soc. Sci.* 1 16–23. 10.21924/chss.1.1.2021.11

[B36] Gómez J. M. Freyd J. J. Delva J. Tracy B. Mackenzie L. N. Ray V. (2023). Institutional courage in action: Racism, sexual violence, & concrete institutional change. *J. Trauma Dissoc.* 24 157–170. 10.1080/15299732.2023.2168245 36744639

[B37] Haynes-Baratz M. C. Metinyurt T. Li Y. L. Gonzales J. Bond M. A. (2021). Bystander training for faculty: A promising approach to tackling microaggressions in the academy. *New Ideas Psychol.* 63:100882. 10.1016/j.newideapsych.2021.100882

[B38] Hellemans C. Dal Cason D. Casini A. (2017). Bystander helping behavior in response to workplace bullying. *Swiss J. Psychol.* 76 135–144. 10.1024/1421-0185/a000200

[B39] Hodgins M. Kane R. Itzkovich Y. Fahie D. (2024). Workplace bullying and harassment in higher education institutions: A scoping review. *Int. J. Environ. Res. Public Health* 21:1173. 10.3390/ijerph21091173 39338057 PMC11431250

[B40] Holm K. Jönsson S. Muhonen T. (2023). How are witnessed workplace bullying and bystander roles related to perceived care quality, work engagement, and turnover intentions in the healthcare sector? A longitudinal study. *Int. J. Nurs. Stud.* 138:104429. 10.1016/j.ijnurstu.2022.104429 36577260

[B41] Hortensius R. de Gelder B. (2018). From empathy to apathy: The bystander effect revisited. *Curr. Direct. Psychol. Sci.* 27 249–256. 10.1177/0963721417749653 30166777 PMC6099971

[B42] Htun M. Jensenius F. R. Dominguez M. S. Tinkler J. Contreras C. (2022). Effects of mandatory sexual misconduct training on university campuses. *Socius* 8:23780231221124574. 10.1177/23780231221124574

[B43] Johnson T. D. Joshi A. (2016). Dark clouds or silver linings? A stigma threat perspective on the implications of an autism diagnosis for workplace well-being. *J. Appl. Psychol.* 101 430–449. 10.1037/apl0000058 26595753

[B44] Jönsson S. Muhonen T. (2022). Factors influencing the behaviour of bystanders to workplace bullying in healthcare — A qualitative descriptive interview study. *Res. Nurs. Health* 45 424–432. 10.1002/nur.22228 35426159 PMC9545846

[B45] Jönsson S. Muhonen T. (2025). Bystander behavior in workplace bullying: A vignette study exploring how organizational space and situational strength influence intentions to intervene. *Int. J. Workplace Health Manag.* 18 331–349. 10.1108/IJWHM-10-2024-0215

[B46] Juvonen J. Nishina A. Graham S. (2000). Peer harassment, psychological adjustment, and school functioning in early adolescence. *J. Educ. Psychol.* 92 349–359. 10.1037/0022-0663.92.2.349

[B47] Keashly L. Neuman J. H. (2010). Faculty experiences with bullying in higher education: Causes, consequences, and management. *Admin. Theory Praxis* 32 48–70. 10.2753/ATP1084-1806320103

[B48] Kelley H. H. (1973). The processes of causal attribution. *Am. Psychol.* 28 107–128. 10.1037/h0034225

[B49] Kettrey H. H. Marx R. A. (2019). The effects of bystander programs on the prevention of sexual assault across the college years: A systematic review and meta-analysis. *J. Youth Adolesc.* 48 212–227. 10.1007/s10964-018-0927-1 30264210

[B50] Khoo S. (2010). Academic mobbing: Hidden health hazard at workplace. *Malays. Fam. Phys.* 5 61–67. PMC4170397 25606190

[B51] Köverová M. Raczova B. (2018). “Negative consequences of helping and the length of work experience,” in *Psychology applications & developments III*, eds Pracana C. Wang M. (Pune: InScience Press), 121–132.

[B52] Latané B. Darley J. M. (1970). *The unresponsive bystander: Why doesn’t he help?.* New York, NY: Appleton-Century-Crofts.

[B53] Leymann H. (1996). The content and development of mobbing at work. *Eur. J. Work Organ. Psychol.* 5 165–184. 10.1080/13594329608414853

[B54] Lin M. Wolke D. Schneider S. Margraf J. (2020). Bullying history and mental health in university students: The mediator roles of social support, personal resilience, and self-efficacy. *Front. Psychiatry* 10:960. 10.3389/fpsyt.2019.00960 31993000 PMC6971115

[B55] Longo J. Sherman R. O. (2007). Leveling horizontal violence. *Nurs. Manag.* 38 34–37, 52-51. 10.1097/01.numa.0000262925.77680.e0. 17491129

[B56] Lund E. M. Ross S. W. (2017). Bullying perpetration, victimization, and demographic differences in college students: A review of the literature. *Trauma Viol. Abuse* 18 348–360. 10.1177/1524838015620818 26759417

[B57] Luo Z. Wang J. Zhou Y. Mao Q. Lang B. Xu S. (2023). Workplace bullying and suicidal ideation and behaviour: A systematic review and meta-analysis. *Public Health* 222 166–174. 10.1016/j.puhe.2023.07.007 37544128

[B58] Lyons M. Brewer G. Gandolfo Conceição M. I. Jaramillo-Sierra A. L. Reyes-Rodriguez M. F. (2025). Barriers and facilitators of bystander intervention in response to racism in Colombia. *Glob. Public Health* 20:2453879. 10.1080/17441692.2025.2453879 39864075

[B59] Machovcová K. Kováts G. Mudrák J. Cidlinská K. Zábrodská K. (2024). (Dis)continuities in academic middle management career trajectories: A longitudinal qualitative study. *J. High. Educ. Pol. Manag.* 46 200–217. 10.1080/1360080X.2023.2276589

[B60] Mahmoudi M. (2019). Academic bullies leave no trace. *Bioimpacts* 9 129–130. 10.15171/bi.2019.17 31508328 PMC6726746

[B61] Mahmoudi M. Keashly L. (2021). Filling the space: A framework for coordinated global actions to diminish academic bullying. *Angewandte Chemie* 133 3378–3384. 10.1002/anie.202009270 33295129

[B62] Manuel P. Tang G. H. Weyand A. James P. Sholzberg M. (2023). Academic bullying in science and medicine: The need for reform. *Res. Pract. Thromb Haemost.* 8:102270. 10.1016/j.rpth.2023.102270 38222079 PMC10784303

[B63] Martinescu E. Janssen O. Nijstad B. A. (2019). Self-evaluative and other-directed emotional and behavioral responses to gossip about the self. *Front. Psychol.* 9:2603. 10.3389/fpsyg.2018.02603 30662417 PMC6328481

[B64] Moore C. (2015). Moral disengagement. *Curr. Opin. Psychol.* 6 199–204. 10.1016/j.copsyc.2015.07.018

[B65] Morrison E. W. Milliken F. J. (2000). Organizational silence: A barrier to change and development in a pluralistic world. *Acad. Manag. Rev.* 25 706–725. 10.2307/259200

[B66] Moss S. E. Mahmoudi M. (2021). STEM the bullying: An empirical investigation of abusive supervision in academic science. *eClinicalMedicine* 40:101121. 10.1016/j.eclinm.2021.101121 34527894 PMC8433114

[B67] Moss S. E. Mahmoudi M. (2024). Examining age-dependent patterns in academic bullying behaviors. *medRxiv [Preprint]* 10.1101/2024.09.24.24314297

[B68] Moss S. Täuber S. Sharifi S. Mahmoudi M. (2022). The need for the development of discipline-specific approaches to address academic bullying. *EClinicalMedicine* 50:101598. 10.1016/j.eclinm.2022.101598 36035438 PMC9403844

[B69] Nadal K. L. Issa M. A. Leon J. Meterko V. Wideman M. Wong Y. (2011). Sexual orientation microaggressions: “Death by a thousand cuts” for lesbian, gay, and bisexual youth. *J. LGBT Youth* 8 234–259. 10.1080/19361653.2011.584204

[B70] Neuman J. H. Baron R. A. (2003). “Social antecedents of bullying: A social interactionist perspective,” in *Bullying and emotional abuse in the workplace: International perspectives in research and practice*, eds Einarsen S. Hoel H. Zapf D. Cooper C. L. (Boca Raton, FL: CRC Press), 185–202.

[B71] Nikolaou D. (2017). Do anti-bullying policies deter in-school bullying victimization? *Int. Rev. Law Econ.* 50 1–6. 10.1016/j.irle.2017.03.001

[B72] Nothling L. (2025). *Review finds harassment and bullying “widespread” at Australian National University’s College of Health and Medicine. ABC News.* Available online at: https://www.abc.net.au/news/2025-05-27/act-anu-health-and-medicine-gender-and-culture-nixon-review/105343268 (accessed June 2, 2025).

[B73] Offer K. Rahwan Z. Hertwig R. (2023). Counteracting deliberate ignorance of academic bullying and harassment. *Commun. Psychol.* 1:38. 10.1038/s44271-023-00044-7 39242959 PMC11332241

[B74] Paull M. Omari M. Standen P. (2012). When is a bystander not a bystander? A typology of the roles of bystanders in workplace bullying. *Asia Pacific J. Hum. Resources* 50 351–366. 10.1111/j.1744-7941.2012.00027.x

[B75] Pinciotti C. M. Orcutt H. K. (2021). Institutional betrayal: Who is most vulnerable? *J. Interpersonal Viol.* 36 5036–5054. 10.1177/0886260518802850 30264672

[B76] Pouwelse M. Mulder R. Mikkelsen E. G. (2021). “The role of bystanders in workplace bullying: An overview of theories and empirical research,” in *Pathways of job-related negative behaviour*, eds D’Cruz P. Noronha E. Baillien E. Catley B. Harlos K. Høgh A. (Berlin: Springer Nature Singapore), 385–422.

[B77] Pozzoli T. Ang R. P. Gini G. (2012). Bystanders’ reactions to bullying: A cross-cultural analysis of personal correlates among Italian and Singaporean students. *Soc. Dev.* 21 686–703. 10.1111/j.1467-9507.2011.00651.x

[B78] Proudhon P. (2025). Cambridge staff unhappy with response to bullying claims. *Bully. Acad. High. Educ.*

[B79] Pyke K. D. (2018). Institutional betrayal: Inequity, discrimination, bullying, and retaliation in academia. *Sociol. Perspect.* 61 5–13. 10.1177/0731121417743816

[B80] Quine L. (1999). Workplace bullying in NHS community trust: Staff questionnaire survey. *BMJ* 318 228–232. 10.1136/bmj.318.7178.228 9915730 PMC27703

[B81] Raboteg-Šarić Z. Bartaković S. (2019). Empathy and moral disengagement as predictors of bystander roles in school bullying. *Central Eur. J. Paediatr.* 15 161–176. 10.5457/p2005-114.248

[B82] Reinholz D. L. Stone-Johnstone A. White I. Sianez L. M. Shah N. (2020). A pandemic crash course: Learning to teach equitably in synchronous online classes. *CBE Life Sci. Educ.* 19:ar60. 10.1187/cbe.20-06-0126 33259278 PMC8693934

[B83] Rivers I. Poteat V. P. Noret N. Ashurst N. (2009). Observing bullying at school: The mental health implications of witness status. *Sch. Psychol. Quar.* 24 211–223. 10.1037/a0018164

[B84] Rudhumbu N. (2015). Managing curriculum change from the middle: How academic middle managers enact their role in higher education. *Int. J. High. Educ.* 4 5958–5958. 10.5430/ijhe.v4n1p106

[B85] Sainio M. Veenstra R. Huitsing G. Salmivalli C. (2010). Victims and their defenders: A dyadic approach. *Int. J. Behav. Dev.* 35 144–151. 10.1177/0165025410378068

[B86] Salin D. (2003). Ways of explaining workplace bullying: A review of enabling, motivating and precipitating structures and processes in the work environment. *Hum. Relations* 56 1213–1232. 10.1177/00187267035610003

[B87] Salmivalli C. (2010). Bullying and the peer group: A review. *Aggress. Viol. Behav.* 15 112–120. 10.1016/j.avb.2009.08.007

[B88] Salmivalli C. Lagerspetz K. Björkqvist K. Österman K. Kaukiainen A. (1996). Bullying as a group process: Participant roles and their relations to social status within the group. *Aggress. Behav.* 22 1–15. 10.1002/(SICI)1098-2337199622:1<1::AID-AB1<3.0.CO;2-T 40370606

[B89] Samuelson W. Zeckhauser R. (1988). Status quo bias in decision making. *J. Risk Uncertainty* 1 7–59.

[B90] Scully M. Rowe M. (2009). Bystander training within organizations. *Journal of the International Ombudsman Association* 2 1–9.

[B91] Sereda T. (2018). *Mapping workplace bullying behaviours to moral disengagement.* Altona: Friesen Press.

[B92] Smith C. P. Freyd J. J. (2013). Dangerous safe havens: Institutional betrayal exacerbates sexual trauma. *J. Traumatic Stress* 26 119–124. 10.1002/jts.21778 23417879

[B93] Smith C. Freyd J. (2014). Institutional betrayal. *Am. Psychol.* 69 575–587. 10.1037/a0037564 25197837

[B94] Song J. Oh I. (2018). Factors influencing bystanders’ behavioral reactions in cyberbullying situations. *Comp. Hum. Behav.* 78 273–282. 10.1016/j.chb.2017.10.008

[B95] Stone D. (2025). *Laissez-faire leadership. In Elgar Encyclopedia of Leadership.* Cheltenham: Edward Elgar Publishing eBooks, 127–128.

[B96] Suler J. (2004). The online disinhibition effect. *Cyberpsychol. Behav.* 7 321–326. 10.1089/1094931041291295 15257832

[B97] Täuber S. Mahmoudi M. (2022). How bullying becomes a career tool. *Nat. Hum. Behav.* 6:475. 10.1038/s41562-022-01311-z 35132170

[B98] Täuber S. Oliveri N. F. Kostakopoulou D. Mahmoudi M. (2022). Breaking the silence around academic harassment. *FEBS Lett.* 596 2337–2344. 10.1002/1873-3468.14473 36052874

[B99] The Guardian (2020). *Third of Cambridge University staff ‘have experienced bullying’.* London: The Guardian.

[B100] Thornberg R. Tenenbaum L. Varjas K. Meyers J. Jungert T. Vanegas G. (2012). Bystander motivation in bullying incidents: To intervene or not to intervene? *West J. Emerg. Med.* 13 247–252. 10.5811/westjem.2012.3.11792 22900122 PMC3415829

[B101] Tight M. (2023). Bullying in higher education: An endemic problem? *Tertiary Educ. Manag.* 29 123–137. 10.1007/s11233-023-09124-z

[B102] Tsang J.-A. (2002). Moral rationalization and the integration of situational factors and psychological processes in immoral behavior. *Rev. General Psychol.* 6 25–50. 10.1037//1089-2680.6.1.25

[B103] Tsuno K. Kawakami N. (2014). Multifactor leadership styles and new exposure to workplace bullying: A six-month prospective study. *Indust. Health* 53 139–151. 10.2486/indhealth.2014-0152 25382384 PMC4380601

[B104] Ulz J. (2022). *How to support a colleague who is a target of academic bullying.* Princeton, NJ: Editage.

[B105] Universities U. K. (2023). *Petition for debate: Creating a statutory legal duty of care for students in higher education* (parliamentary briefing). London: Universities UK.

[B106] Vaill Z. Campbell M. Whiteford C. (2023). University students’ knowledge and views on their institutions’ anti-bullying policy. *High. Educ. Pol.* 36 1–19. 10.1057/s41307-021-00244-y

[B107] Valdez J. W. Sharma S. Gould J. (2025). Systemic territoriality in academia: The Gollum effect’s impact on scientific research and careers. *One Earth* 8:101314. 10.1016/j.oneear.2025.101314

[B108] Xie Z. Liu C. Teng Z. (2023). The effect of everyday moral sensitivity on bullying bystander behavior: Parallel mediating roles of empathy and moral disengagement. *J. Interpersonal Viol.* 38 7678–7701. 10.1177/08862605221147071 36636877

[B109] Zabrodska K. Linnell S. Laws C. Davies B. (2011). Bullying as intra-active process in neoliberal universities. *Qual. Inquiry* 17 709–719. 10.1177/1077800411420668

[B110] Zhang Z. Song P. (2020). Multi-level effects of humble leadership on employees’ work well-being: The roles of psychological safety and error management climate. *Front. Psychol.* 11:571840. 10.3389/fpsyg.2020.571840 33262726 PMC7685992

